# Preliminary Normative Data of Persian Phonemic and Semantic Verbal Fluency Test

**Published:** 2018-10

**Authors:** Ensiyeh Ghasemian-Shirvan, Saba Molavi Shirazi, Masoume Aminikhoo, Mostafa Zareaan, Hamed Ekhtiari

**Affiliations:** 1 Translational Neuroscience Program, Institute for Cognitive Science Studies, Tehran, Iran.; 2 Department of Psychology, Shahid Beheshti University, Tehran, Iran.; 3 Department of Psychology, Allameh Tabataba'i University, Tehran, Iran.; 4 Neurocognitive Laboratory, Iranian National Center for Addiction Studies, Tehran University of Medical Sciences, Tehran, Iran.

**Keywords:** *Farsi*, *Neuropsychological Assessment*, *Normative Data*, *Persian*, *Verbal Fluency*

## Abstract

**Objective:** Verbal fluency tests (VFTs) are widely used in clinical practice and research to assess executive functions and are highly sensitive to frontal lobe lesions. However, using VFTs in different cultures and languages needs further considerations. The aim of this study was to provide a Persian (Farsi) version of verbal fluency with preliminary normative data.

**Method** **:** In the first phase, 50 healthy native Persian-speaking individuals completed 1-minute VFT for all 32 letters in Persian to find letters with highest frequency. In the second phase, 100 healthy participants (50 females) were recruited into 5 age groups that were matched by gender and education. Participants were instructed to do 1-minute VFT for the 3 selected letters (phonemic VFT) and 3 categories (animal, supermarket, and fruit) (semantic VFT). For data analysis, one-way ANOVA was performed.

**Results: **In the first phase, 3 letters (Pe standing for /P/, Meem for /M/ and Kaaf for /K/) had the highest frequency in word production (12 in average) and had been chosen for Persian phonemic VFT. Participants were assessed with the 3 selected letters (/P/: 12.28±3.607, /M/: 12.54±3.907, and /K/: 12.48±3.708) and 3 semantic categories (animal: 21.67±5.119, supermarket: 21.19±4.907, and fruit: 19.58±4.439) with 1-minute time limitation for each test. The results showed that education was significantly (p<0.01) associated with the performance in the phonemic but not semantic scores, while age was not correlated with either of the tests. No significant effect of gender was observed.

**Conclusion: **Based on our results, we recommend Persian letters Pe, Meem, and Kaff that have the highest frequency in word production among others to be used for neuropsychological assessments and future studies in the Persian language. This is the same logic behind selecting F, A, and S in the English version. Although the norms obtained in this study are preliminary, these results can be useful in clinical evaluation with considerations about age and educational levels. Moreover, the findings of this study can be used as an initial step for more comprehensive normative studies.

Verbal fluency tests (VFTs) have an important role in neuropsychological and clinical research and are included in almost every neuropsychological evaluation (1, 2). The importance of VFTs in medicine comes from the fact that they can be utilized as rapid measures to assess prefrontal cortex functions and detect cognitive impairments from a variety of etiologies, especially dementias (3, 4, 5 and 6). VFTs are sensitive to frontal lobe lesions, particularly lesions in the left frontal lobe (7), and they are also sensitive to lesions in the temporal lobe and caudate nucleus (8).

VFTs measure retrieval speed of words under specific rules in a limited time. There are 2 types of verbal fluency tasks: (1) semantic or categorical; (2) phonemic or letter fluency. In semantic VFTs, participants are required to say as many words as they can from a specific category. 

The most common category is “animals”, but other categories such as “fruits and vegetables”, “things found in the kitchen”, “things to wear”, “things found in a supermarket”, and “vehicles” have also been used in some studies. In phonemic VFTs, participants are required to produce as many words as possible beginning with a specific letter. There are usually 3 letters in phonemic fluency tasks as there was in the original English version. F, A, and S are the most commonly used letters in English5, and other letter combinations, including C, F, L, P, R, and W, are also used (9, 10). These sets of letters are widely used in English and even in some other languages; however, they should be used with caution in other languages due to different characteristics of each language. The choice of the letter set can affect the results to some extent because of the differences in word frequency and letter difficulties for each letter in different languages (5).

The necessity of language consideration for semantic fluency may be less obvious. Yet, many studies showed significant differences between speakers of different languages in semantic fluency in different categories that were not explained either by age and/or education or by vocabulary size (5, 11). For example, verbal fluency score for animal category varies in different languages, including Chinese (12), Arabic (13), Italian (14), Spanish (15), Hindi (16) and Hebrew (2). In a direct comparison, Kempler et al. (1998) found significant differences between the performance of Spanish and Vietnamese immigrants in the United States. Spanish speakers produced fewer words in the category of animals compared to Vietnamese and English speakers, whereas the Vietnamese got higher scores in animal verbal fluency. Researchers attribute the difference to the length of animal names in each language. According to the study, Vietnamese animal names were mostly monosyllabic, but Spanish animal names were mostly multisyllabic (11). These findings highlight the importance of considering the differences between languages and cultures when using verbal fluency tasks (10). Accordingly, normative data for Neo- Latin languages in general and English language in particular are inapplicable to other languages with different characteristics, such as Persian (Farsi). To date, to our knowledge, only 3 studies have been published on VFTs in Persian speaking populations, and none of them has provided any letter selection process or any normative data for healthy adults. 

In an attempt to produce normative data for bilingual adolescent, Malek et al. (2013) developed a VFT for 302 bilingual (Turkish-Persian) adolescents (11-18) in Tabriz, Iran17. Two other studies were conducted on children and adolescents with developmental stuttering and non-demented patients with Parkinson’s disease. They’ve both used Persian analogues for the letters F, A, and S without any letter selection process (3, 18). 

Thus, if we want to use any language and/or culture bounded neuropsychological test out of the context they have been developed in, it is important to take necessary developmental steps to modify the tasks to suit the target culture and language and provide adequate norms considering demographic factors.

Most of the published studies support the effects of demographic factors, mainly age, education, and gender, on verbal fluency tasks in both phonemic and semantic versions. Considering different studies with different age ranges, a nonlinear effect of age is apparent in both phonemic and semantic fluency. In phonemic fluency tasks, performance improves during childhood, and the scores increase from early childhood to adulthood. The increase peaks at about age 30 to 39 and shows a mild decline in older ages (2, 5, 19, 20, 21, 22 and 34). Semantic fluency scores follow the same pattern although it starts to show a mild decline from about age 20 (5, 22) and it is more strongly related to age than phonemic fluency scores (5, 23, 24, 25).

Published reports for the influence of education levels on both phonemic and semantic fluency tasks are even more consistent. Higher level of education is associated with better performance on both tasks (2, 5, 10, 11, 13, 20, 21, 22, 24, 26, 27, 28, 29, 30, 31, 32 and 33). Crossly et al. (1977) found that healthy older participants with highest educational level (i.e., 13 or more years) generated more than twice as many words as those with lowest educational level (i.e., less than 6 years). In a study on a Greek sample, Kosmidis et al. (2004) reported that education was the most influential demographic factor in both semantic and phonemic fluency tasks. Although educational level effects have been emphasized by many studies, they are not always the best predictor of performance. Kave et al. (2005) reported that among Hebrew speakers, age was the best predictor for both semantic and phonemic fluency scores.

The other demographic characteristic, which should be considered in the VFTs, is gender. Many studies found little or no evidence for gender effects on phonemic and semantic scores (2, 13, 27, 28, 30, 33 and 34). Although there are few reports that indicate females may slightly outperform men on phonemic fluency tests (17, 21, 32), the effects of gender on fluency performance is observed in just few certain semantic categories, not all of them (14, 33, 35). However, when this variable is considered together with age and educational level, the latter has a much greater effect on fluency than gender does.

Today, Persian is spoken primarily in Iran and Afghanistan, but it is also spoken by significant populations in other countries around the Persian Gulf (Bahrain, Iraq, Oman, People's Democratic Republic of Yemen, and the United Arab Emirates) as well as by large communities in the USA. According to Penn language center (38), Persian is the mother tongue for about 50% of Iran's population and it is the official language of the country, with the population of 80 million. There are also over 5.5 million Persian speakers in Afghanistan (25% of the population) and about 1 million Persian speakers in Pakistan. The aim of the present study was to establish an appropriate version of phonemic and semantic fluency for Persian speaking communities and provide preliminary norms for these tests for Persian speaking adult population in different ages, educational levels, and gender groups.

## Materials and Methods

In this study, we aimed to developa Persian version of verbal fluency tasks and provide preliminary norms for it. To reach the mentioned goal, the study comprised of 2 phases: (1) Development phase; (2) Standardization phase.

The sample for the first phase consisted of 47 healthy participants (25 males, 22 females, Mean age 32.21 years ± 9.77; mean of educational level 12.19 ± 3.77 years). Participants were recruited based on the following criteria:

1. Age 18-60 years
2. Being Iranian and Persian native speaker as their first mother tongue
3. Having no medical disease that interferes with the assessments and no psychiatric disorder in both axis I and II of DSM-IV-TR or any other neurological or speech problems based on a structured clinical interview

In the second phase, 100 healthy individuals (50 males, 50 females) were classified into 5 age groups (15–24, 25–34, 35–44, 45–54, and 55–65 years.), and 2 groups according to educational level (high school diploma, Bachelor of science/art) in a balanced way in 20 clusters (gender*age*education level) with 5 participants each. The inclusion criteria were the same as the first phase. All participants were given informed consent forms, which included study conditions and benefits. Also, they were compensated for their time in the study with small gifts.

Materials and Procedure 

In the first phase of this study, after a thorough instruction based on a written text and doing a sample trial to familiarize the participants with the test, they were asked to name as many words as they could, starting with specific letters in 60 seconds. Participants performed the task for all 32 letters in the Persian alphabet plus a specific vowel “آ” (aka Aa). The order of presenting letters was randomized to control the effects of learning and tiredness over different letters. Participants were instructed that proper nouns and multiple words using the same stem with a different suffix (e.x. suffixes to make plural nouns of possessive pronouns) were not acceptable. Also, the same verb conjugated in different tenses were not acceptable and counted as one. When a questionable response was provided, clarifications were made by the instructor.

Time was measured by a stopwatch and participants’ answers were recorded. Final score for each letter was calculated after omitting errors and duplicates. Instructions were as follow: “I want you to say as many Persian words as possible that begin with a certain letter. You can say any word except for names of people and other proper nouns, such as Sarahor Tehran. Also, you should use different words rather than the same word with a different ending. For example, different forms of a verb or single and plural forms of a noun are not acceptable.” According to the results of the first phase, 3 letters (Pe standing for /P/, Meem for /M/, and Kaaf for /K/) were selected for the Persian version of phonemic fluency test. These letters were selected because they had the highest frequency in world production among others (Table 1), following the same logic that is behind selecting F, A, and S in the English version. 

The same procedure of administration and scoring were used for these 3 selected letters in the second phase to get a preliminary normative data among 100 participants. To measure semantic fluency in the second phase, 3 categories, “animals”, “fruits”, and “things found in the supermarket”, were presented to participants in random order. Instructions were as follow: “I want you to say as many Persian words as possible that belong to a certain category. You should use different words rather than the same word with a different ending. For example, single and plural forms of a noun are not acceptable.” 

For comprising the data, we used one-way ANOVA to compare age-related and educational factors for producing words.

^1^ Sara

^2^ Tehran

## Results

The mean frequency of words produced in 60 seconds during the first phase of the study is shown in Table 1. The letters Pe, Meem, and Kaaf have the highest numbers of generated words with a mean of over 12 words produced for each of them.

In the second phase, the total correct answers for the phonemic test with 3 letters and semantic test with 3 categories were calculated for statistical analysis. The mean score on the phonemic and semantic fluency for the whole sample was 37.30±9.58 and 62.44±11.89. Mean scores and standard deviations of phonemic and semantic fluency are presented according to different age groups, education level, and gender in Tables 2 and 3.

The normality of sample was verified using the Kolmogorov-Smirnov test (K-S). One-way ANOVA results showed that phonemic fluency is significantly influenced by education (F (1, 99) = 11.46, p =.001), but not by age groups (F (4, 99) = 0.73, p = 0.593) or gender (F (1, 99) = 0.66, p = 0.419). No interaction was observed between any of the variables tested by three-way ANOVA (Table 4). No variable showed a significant effect in the semantic fluency test (Table 5).

Correlation analyses indicated that performance on the 2 components of the fluency test (ie, phonemics and semantics) was highly correlated (**P**= 0.438; P = .000). In addition, all measures of phonemic fluency performance were positively correlated with education level; moreover, a weak but significant positive correlation was observed between animal fluency and education (**P**= 0.215, P = 0.032) (Table 6).

**Table 1:** Mean Frequency for Produced Words for ach Letter in 60 Seconds without Considering Integers for each Letter’s Value

**Letters**		**Mean**	
P^1^ M^2^ K^3^ KH^4^ D^5^ B^6^ SH^7^ T^8^ R^9^ N^10^ J^11^ S^12^ G^13^ A^14^ A ^15^ F^16^ CH^17^ Z^18^ GH^19^ L^20^ E^21^ V^22^ H^23^ H^24^ Y^25^ T^26^ S^27^ GH^28^ J^29^ Z^30^ S^31^ Z^32^ Z^33^		12 11 10 9 8 6 5 4 3 2 1	
1. Pe 2. Meem 3. Kaff 4. Khe 5. De 6. Be 7. She 8. Te 9. Re 10. Noon 11. Je	12. Sin 13. Gaff 14. Alef 15. Aa 16. Fe 17. Che 18. Ze 19. Ghaaf 20. Laam 21. Eien 22. Vaav		23. He 24. Ha 25. Ye 26. Taa 27. Saad 28. Ghein 29. Czhe 30. Zaa 31. Se 32. Zaad 33. Ze

**Table 2:** Scores of Phonemic Fluency Distributed by Age, Educational Level, and Gender

		**P**		**Meem**		**Kaaf**		**Total**	
	**n**	**M**	**SD**	**M**	**SD**	**M**	**SD**	**M**	**SD**
Age									
15 - 24	20	11.65	4.056	13.00	3.713	12.40	3.858	37.05	10.410
25 - 34	20	11.80	2.648	11.80	3.503	11.60	3.347	35.20	7.777
35 - 44	20	12.05	3.236	11.85	3.703	12.10	3.684	36.00	8.687
45 - 54	20	13.00	4.507	12.65	4.380	13.15	4.545	38.80	12.042
55 – 65	20	12.90	3.447	13.40	4.297	13.15	3.066	39.45	8.696
Education									
Diploma	50	11.34	3.623	11.26	3.602	11.60	3.283	34.20	8.797
Bachelor	50	13.22	3.370	13.82	3.810	13.36	3.927	40.40	9.413
Gender									
Male	50	12.24	3.640	12.28	4.314	12.00	4.170	36.52	10.628
Female	50	12.32	3.611	12.80	3.476	12.96	3.149	38.08	8.451

**Table 3:** Scores of Semantic Fluency Distributed by Age, Educational Level, and Gender

		**Animal**		**Supermarket**		**Fruit**		**Total**	
	**n**	**M**	**SD**	**M**	**SD**	**M**	**SD**	**M**	**SD**
Age									
15 - 24	20	20.90	5.647	19.65	5.174	18.55	3.663	59.10	12.993
25 - 34	20	20.35	6.209	20.85	5.687	19.20	5.307	60.40	14.608
35 - 44	20	21.75	4.756	20.75	4.723	18.90	3.110	61.40	9.058
45 - 54	20	23.30	4.244	22.70	4.293	21.55	5.624	67.55	11.450
55 – 65	20	22.05	4.478	22.00	4.437	19.70	3.729	63.75	9.711
Education									
Diploma	50	20.70	4.523	21.18	4.835	19.24	4.104	61.12	10.590
Bachelor	50	22.64	5.528	21.20	5.026	19.92	4.767	63.76	13.030
Gender									
Male	50	21.58	5.444	20.72	4.907	19.04	4.594	61.34	12.351
Female	50	21.76	4.826	21.66	4.910	20.12	4.255	63.54	11.422
Total	100	21.67	5.119	21.19	4.907	19.58	4.439	62.44	11.887

**Table 4:** Three-Way ANOVA among Phonemic Fluency, Age groups, Education Level, and Gender

**Source**	**Df**	**F**	**Sig.**
Education	1	11.659	0.001
Age Group	4	0.791	0.535
Gender	1	0.738	0.393
Education * Age Group	4	0.994	0.416
Education * Gender	1	0.012	0.913
Age Group * Gender	4	1.502	0.209
Education * Age Group * Gender	4	1.197	0.319
Error	80		

**Table 5:** Three-Way ANOVA among Semantic Fluency, Age Groups, Education Levels, and Gender

**Source**	**Df**	**F**	**Sig.**
Education	1	1.252	0.267
Age Group	4	1.589	0.185
Gender	1	0.869	0.354
Education * Age Group	4	0.193	0.941
Education * Gender	1	0.023	0.879
Age Group * Gender	4	1.184	0.324
Education * Age Group * Gender	4	1.622	0.177
Error	80		

**Table 6:** Spearman Correlation between Fluency Scores and Age and Education

	**/P/**	**/M/**	**/K/**	**Phonemic Score**	**Animal**	**Supermarket**	**Fruit**	**Semantic Score**
/P/								
/M/	0.55^a^							
/K/	0.55^a^	0.66^a^						
Phonemic Score	0.80^a^	0.87^a^	0.87^a^					
Animal	0.33^a^	0.30^a^	0.50^a^	0.44^a^				
Supermarket	0.22^a^	0.18	0.28^a^	0.28^a^	0.46^a^			
Fruits	0.28^a^	0.24^b^	0.38^a^	0.35^a^	0.50^a^	0.56^a^		
Semantic Score	0.36^a^	0.30^a^	0.47^a^	0.44^a^	0.79^a^	0.82^a^	0.81^a^	
Education	0.26^a^	0.35^a^	0.27^a^	0.35^a^	0.21^b^	0.01	0.07	0.11
Age Group	0.13	0.08	0.14	0.12	0.13	0.19	0.14	0.18

a. Correlation is significant at 0.01 level.

b. Correlation is significant at 0.05 level.

## Discussion

The main objective of this study was to develop a verbal fluency test for a Persian speaking population and obtain preliminary normative data for healthy Iranian adults. The only existing norms for verbal fluency in Persian have used a restricted age range (i.e., 11-18) and ethnic group and were not suitable to be used for healthy adults (17).

The current findings showed that education level is the only correlate of performance on phonemic fluency tests among basic demographic measures, as seen in other studies as well (10, 36). Meanwhile, in contrast with previous studies in which education had a significant effect on semantic fluency, no significant relationship was observed between education and semantic score (12). This could be due to the limitation of our study, which included only two educational levels and did not consist of individuals with very high or very low education.

Education level was positively correlated with the phonemic score and its components, and a weak but significant relationship was observed between education level and animals’ category, but not other categories. The results indicated that the lowest education level was 12 years and the highest was 16 years. Education is still an important factor in generating words with specific letters. This finding is in contrast with a previous study )^2^( which reported that the great effect of education is due to the inclusion of people with very low educational level and it further indicated that when a study is conducted with more educated individuals, education will not a predictor for semantic fluency anymore.

Evidence from a variety of sources shows that verbal fluency measures are relatively insensitive to gender (2, 12, 14, 27, 28, 30, 33, 35, 37). The results obtained in our study supported these reports, moreover, no significant gender effect was observed in fluency performance for either tasks.

According to the results of this study, there was no significant difference between age groups in their verbal fluency. These results conform to some of the previous studies that reported non-significant age effects (30, 36). However, they are different from many other studies that showed a downward trend in verbal fluency with increase in age (35, 37). This can be because of the demographic characteristic of our sample; our oldest participant was 60 years old. Nevertheless, most of the studies that reported a decrease in word production in association with age included older participants. The study by Tombaugh et al. (1999) supports this assumption. They reported that although in fluency category (animal naming), age accounted for 23.4% of the variance, the number of animals named remained relatively constant until the age of 60 and, then, it began to decrease by increase in age. 

Two major factors should be kept in mind when using the current norms. First, the norms are applicable only when these letters and categories are used. A degree of variety may exist among the number of words generated for different letters or categories. Second, although the formal language in Iran is Persian (Farsi), Iranian population consists of monolinguals and bilinguals, and in many ethnic groups in different regions of Iran, and the first language might not be the formal language. This difference may affect performance in verbal fluency for these groups and should be considered when conducting the tests in such regions and also in rural regions. 

## Limitation

One of the limitations of this study was the relative low number of participants from five different age groups with two different educational levels. Also, we did not assess other cognitive functions or IQ of the participants. Furthermore, all participants were selected from healthy population and we assumed that they had normal cognitive function.

## Conclusion

Taken together, to use a culture and language bound test such as verbal fluency in speakers of different languages, these tests must be modified and standardized in the target language under thorough examination. In this study, we selected letters in phonemic fluency task to suit our plan and provide primary normative data for healthy adults for the first time. We recommend Persian letters Pe, Meem, and Kaff that have the highest frequency in word production among others to be used for neuropsychological assessments and future studies in the Persian language. This is the same logic behind selecting F, A, and S in the English version. Likewise, our results showed the effect of education on phonemic tasks and revealed no age or sex effect on performance within this age range. The given data are expected to assist neuropsychologists and clinicians in evaluating adult Iranian patients and may help more confident clinical diagnoses.

## References

[1] Lezak MD (2004). Neuropsychological assessment.

[2] Kavé G (2005). Phonemic fluency, semantic fluency, and difference scores: Normative data for adult Hebrew speakers. J Clin Exp Neuropsychol.

[3] Dadgar H, Khatoonabadi AR, Bakhtiyari J (2013). Verbal Fluency Performance in Patients with Non-demented Parkinson’s Disease. Iran J Psychiatry.

[4] Henry JD, Crawford JR, Phillips LH (2004). Verbal fluency performance in dementia of the Alzheimer’s type: a meta-analysis. Neuropsychologia.

[5] Strauss E, Sherman EM, Spreen O (2006). A compendium of neuropsychological tests: Administration, norms, and commentary.

[6] Henry JD, Crawford JR (2004). A meta-analytic review of verbal fluency performance following focal cortical lesions. Neuropsychology.

[7] Tombaugh TN, Kozak J, Rees L (1999). Normative Data Stratified by Age and Education for Two Measures of Verbal Fluency: FAS and Animal Naming. Arch Clin Neuropsychol..

[8] Benton AL, Hamsher KD, Sivan AB (1994). Multilingual aphasia examination: manual of instructions. AJA Assoc.

[9] Ruff RM, Light RH, Parker SB, Levin HS (1996). Benton controlled oral word association test: Reliability and updated norms. Arch Clin Neuropsychol.

[10] Kempler D, Teng EL, Dick M, Taussig I, Davis DS (998). The effects of age, education, and ethnicity on verbal fluency. J Int Neuropsychol Soc.

[11] Chan AS, Poon MW (1999). Performance of 7-to 95-year-old individuals in a Chinese version of the category fluency test. J Int Neuropsychol Soc.

[12] Khalil MS (2010). Preliminary Arabic normative data of neuropsychological tests: The verbal and design fluency. J Clin Exp Neuropsychol.

[13] Capitani E, Laiacona M, Barbarotto R (1999). Gender affects word retrieval of certain categories in semantic fluency tasks. Cortex.

[14] Benito-Cuadrado MM, Esteba-Castillo S, Böhm P, Cejudo-Bolivar J, Peña-Casanova J (2002). Semantic verbal fluency of animals: a normative and predictive study in a Spanish population. J Clin Exp Neuropsychol.

[15] Ratcliff G, Ganguli M, Chandra V, Sharma S, Belle S, Seaberg E (1998). Effects of literacy and education on measures of word fluency. Brain Lang.

[16] Malek A, Hekmati I, Amiri S, Pirzadeh J, Gholizadeh H (2013). Designing and standardization of Persian version of verbal fluency test among Iranian bilingual (Turkish-Persian) adolescents. J Anal Res Clin Med.

[17] Bahrami H, Nejati V, Pooretemad H (2014). A Comparative Study of Phonemic and Semantic Verbal Fluency in Children and Adolescents with Developmental Stuttering. Zahedan J Res Med Sci.

[18] Gladsjo JA, Schuman CC, Evans JD, Peavy GM, Miller SW, Heaton RK (1999). Norms for letter and category fluency: Demographic corrections for age, education, and ethnicity. Assessment.

[19] Heaton RK (2004). Heaton RK. Revised comprehensive norms for an expanded Halstead-Reitan Battery: Demographically adjusted neuropsychological norms for African American and Caucasian adults, professional manual.

[20] Loonstra AS, Tarlow AR, Sellers AH (2001). COWAT metanorms across age, education, and gender. Appl Neuropsychol.

[21] Mitrushina M (2005). Handbook of normative data for neuropsychological assessment.

[22] Brickman AM, Paul RH, Cohen RA, Williams LM, MacGregor KL, Jefferson AL (2005). Category and letter verbal fluency across the adult lifespan: relationship to EEG theta power. Arch Clin Neuropsychol.

[23] Mathuranath PS, George A, Cherian PJ, Alexander A, Sarma SG, Sarma PS (2003). Effects of age, education and gender on verbal fluency. J Clin Exp Neuropsychol..

[24] Peña-Casanova J, Quiñones-Úbeda S, Gramunt-Fombuena N, Quintana-Aparicio M, Aguilar M, Badenes D (2009). Spanish Multicenter Normative Studies (NEURONORMA Project): norms for verbal fluency tests. Arch Clin Neuropsychol.

[25] Acevedo A, Loewenstein DA, Barker WW, Harwood DG, Luis C, Bravo M (2000). Category fluency test: normative data for English-and Spanish-speaking elderly. J Int Neuropsychol Soc..

[26] Anstey KJ, Matters B, Brown AK, Lord SR (2000). Normative data on neuropsychological tests for very old adults living in retirement villages and hostels. Clin Neuropsychol.

[27] Bäckman L, Wahlin \AAke, Small BJ, Herlitz A, Winblad B, Fratiglioni L (2004). Cognitive functioning in aging and dementia: The Kungsholmen Project. Aging Neuropsychol Cogn.

[28] Gladsjo JA, Schuman CC, Evans JD, Peavy GM, Miller SW, Heaton RK (1999). Norms for letter and category fluency: demographic corrections for age, education, and ethnicity. Assessment.

[29] Al-Ghatani AM, Obonsawin MC, Al Moutaery KR (2009). Normative data for the two equivalent forms of the Arabic verbal fluency test. Pan Arab J Neurosurg.

[30] Casals-Coll M, Sánchez-Benavides G, Quintana M, Manero RM, Rognoni T, Calvo L (2013). Spanish normative studies in young adults (< i> NEURONORMA</i> young adults project): Norms for verbal fluency tests. Neurologia.

[31] Crossley M, D’arcy C, Rawson NS (1997). Letter and category fluency in community-dwelling Canadian seniors: A comparison of normal participants to those with dementia of the Alzheimer or vascular type. J Clin Exp Neuropsychol.

[32] Machado TH, Fichman HC, Santos EL, Carvalho VA, Fialho PP, Koenig AM (2009). Normative data for healthy elderly on the phonemic verbal fluency task-FAS. Dement Neuropsychol.

[33] KOSMIDIS MH, Vlahou CH, Panagiotaki P, Kiosseoglou G (2004). The verbal fluency task in the Greek population: Normative data, and clustering and switching strategies. J Int Neuropsychol Soc.

[34] Laws KR (1999). Gender affects naming latencies for living and nonliving things: Implications for familiarity. Cortex.

[35] Tombaugh TN, Kozak J, Rees L (1999). Normative data stratified by age and education for two measures of verbal fluency: FAS and animal naming. Arch Clin Neuropsychol.

[36] Anderson P, Northam E, Jacobs R, Catroppa C (2001). Development of executive functions through late childhood and adolescence in an Australian sample. Dev Neuropsychol.

[37] Bolla KI, Lindgren KN, Bonaccorsy C, Bleecker ML (1990). Predictors of verbal fluency (FAS) in the healthy elderly. J Clin Psychol.

[38] Assefi Shirazi T [Penn Language Center].

